# A Concept for Preoperative and Intraoperative Molecular Imaging and Detection for Assessing Extent of Disease of Solid Tumors

**DOI:** 10.3389/or.2024.1409410

**Published:** 2024-07-25

**Authors:** Charles L. Hitchcock, Gregg J. Chapman, Cathy M. Mojzisik, Jerry K. Mueller, Edward W. Martin

**Affiliations:** ^1^ Department of Pathology, College of Medicine, The Ohio State University, Columbus, OH, United States; ^2^ Actis Medical, LLC, Powell, OH, United States; ^3^ Department of Electrical and Computer Engineering, College of Engineering, The Ohio State University, Columbus, OH, United States; ^4^ Division of Surgical Oncology, Department of Surgery, College of Medicine, The Ohio State University, Columbus, OH, United States

**Keywords:** radionuclide, fluorescence, guided-surgery, intraoperative-imaging, fluorescence-guided surgery

## Abstract

The authors propose a concept of “systems engineering,” the approach to assessing the extent of diseased tissue (EODT) in solid tumors. We modeled the proof of this concept based on our clinical experience with colorectal carcinoma (CRC) and gastrinoma that included short and long-term survival data of CRC patients. This concept, applicable to various solid tumors, combines resources from surgery, nuclear medicine, radiology, pathology, and oncology needed for preoperative and intraoperative assessments of a patient’s EODT. The concept begins with a patient presenting with biopsy-proven cancer. An appropriate preferential locator (PL) is a molecule that preferentially binds to a cancer-related molecular target (i.e., tumor marker) lacking in non-malignant tissue and is the essential element. Detecting the PL after an intravenous injection requires the PL labeling with an appropriate tracer radionuclide, a fluoroprobe, or both. Preoperative imaging of the tracer’s signal requires molecular imaging modalities alone or in combination with computerized tomography (CT). These include positron emission tomography (PET), PET/CT, single-photon emission computed tomography (SPECT), SPECT/CT for preoperative imaging, gamma cameras for intraoperative imaging, and gamma-detecting probes for precise localization. Similarly, fluorescent-labeled PLs require appropriate cameras and probes. This approach provides the surgeon with real-time information needed for R0 resection.

## Introduction

Surgery remains the primary curative treatment for colorectal carcinoma (CRC) and other solid tumors. Hall and Ruutiainen [1] stated that in the case of CRC, “complete excision of all areas of disease provides the only curative treatment of all stages of localized disease (stages I-III), and stage IV disease with limited liver and lung metastases.” This article goes beyond routine pathology findings. We refer to “disease-involved tissue” (DIT) as tissue that may or may not contain apparent metastatic tumor cells on routine histopathologic examination; however, this tissue binds the PL, which is demonstrated on molecular imaging or probing, thus indicating disease involvement. Our experience demonstrates that incomplete identification of DIT leads to clinical decisions that can result in unresected disease, a significant cause of morbidity and mortality [2, 3]. The tissue left behind after cytoreductive surgery may be immunocompromised. In the case of CRC, recurrence usually occurs within 2 years of primary surgery, and distant recurrence sites vary between colon and rectal tumors. In contrast, local recurrences occur at the margins and in unresected lymph nodes associated with aberrant lymphatics and lymph nodes outside the anatomic planes of resection [3–5]. An accurate, real-time EODT assessment will optimize intraoperative treatment decisions, thereby reducing the incidence of recurrence while improving patient survival.

## Hypothesis and Theory: Proof of Concept

### The Concept

**FIGURE 1:**
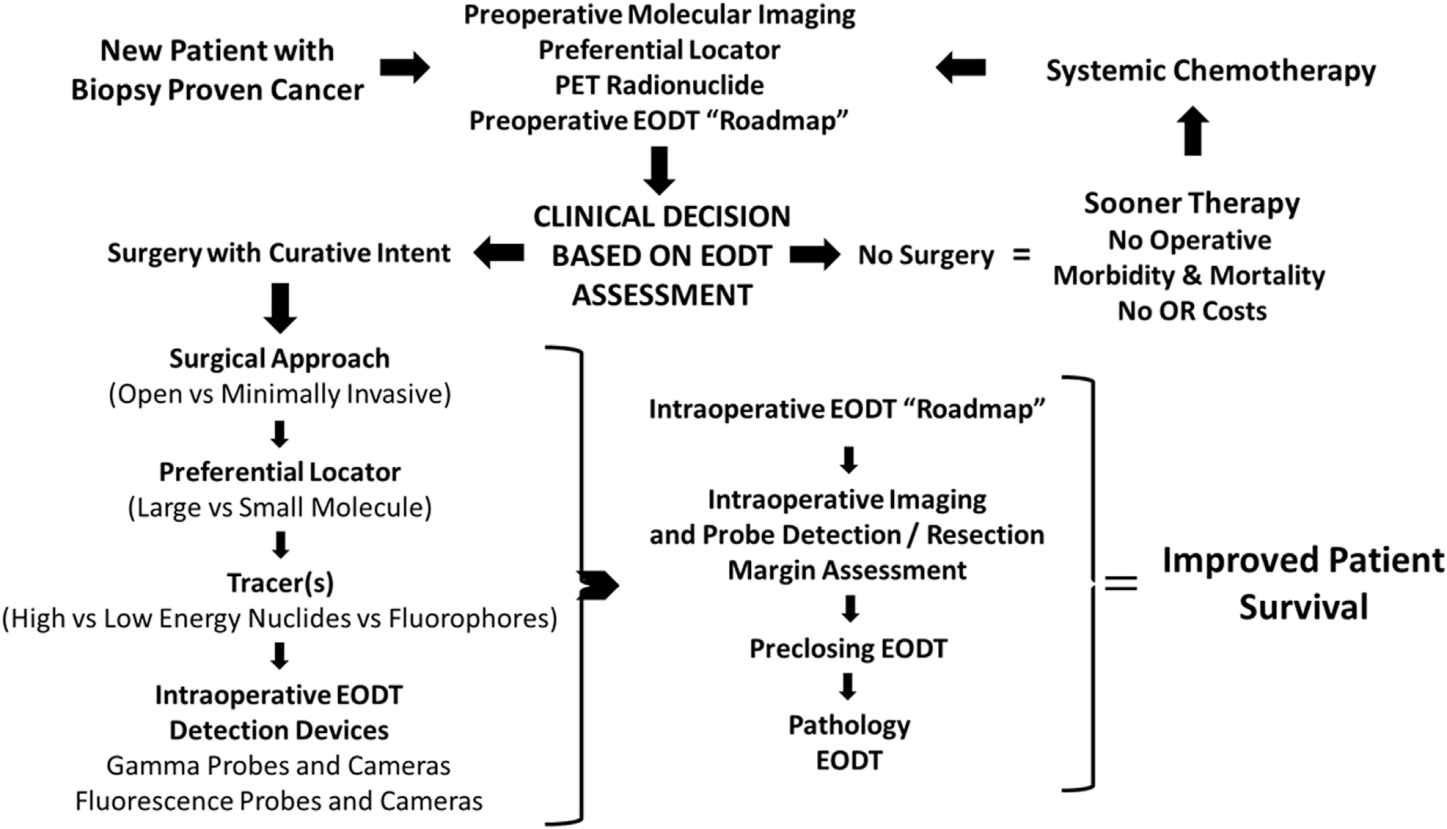
Concept- Assessing the Extent of Diseased Tissue (EODT) in Solid Tumors. As an example, a patient with a biopsy-proven solid tumor prostate carcinoma undergoes preoperative molecular imaging (e.g., PET/CT scan) after injection of a radiolabeled preferential locator (e.g., ^68^Ga-PSMA-11) to assess the extent of diseased tissue (EODT). The EODT assessment will yield a go-to-surgery or no-surgery treatment decision. If there is no surgery, systemic therapy will be administered promptly without postoperative delays, morbidity, or mortality associated with a surgical procedure. During a surgical procedure, intraoperative imaging and a gamma probe will provide real-time EODT information to aid the surgeon in excising disease-involved tissues (DIT). A final image needs to be obtained before closure to confirm the complete excision of DIT. The anticipated outcome is improved patient survival.

Using our experience with colorectal carcinoma and, more recently, neuroendocrine carcinomas at The Ohio State University Wexner Medical Center over the past four decades, the authors propose a “systems engineering” concept for assessing the EODT in solid tumors. Figure 1 depicts a model for this concept. This model, applicable to various solid tumors, combines resources from surgery, nuclear medicine, radiology, pathology, and oncology needed for preoperative and intraoperative assessments of a patient’s EODT. The model begins with a patient presenting to the team with biopsy-proven cancer. The concept’s essential element is a preferential locator (PL), a targeting molecule that preferentially binds to specific tumor-associated molecules not expressed in non-malignant tissues. PL detection requires labeling with an appropriate tracer radionuclide, a fluoroprobe, or both. Preoperative imaging and intraoperative imaging and detection of a radionuclide tracer’s signal utilize a wide variety of modalities (e.g., PET, PET/CT, SPECT, SPECT/CT, portable gamma cameras, and gamma-detecting probes), and fluorescent cameras and probes use fluorochrome-labeled PLs. However, the inherent variability of each imaging and detection technique is still present. In addition, the concept addresses the impact on the patient when a “No Surgery” clinical decision is made. The “No Surgery” decision allows the patient to undergo medical therapy immediately without treatment delays relative to postoperative recovery and eliminates any morbidity and mortality associated with a non-curative surgery. The “no surgery” clinical decision is not a final one. Molecular reimaging to assess for the EODT should be a critical part of the follow-up for patients receiving systemic neoadjuvant chemotherapy in order to balance “the need for radical removal of disease and minimizing the scope of surgery.” [6]

Initial EODT assessment requires preoperative hybrid molecular imaging (e.g., PET/CT or SPECT/CT) to provide a “roadmap” of the locations of the DIT that correlates both the anatomic and molecular features of the tumor. However, this correlation depends on the PL the multidisciplinary team members selected [7, 8]. Using the EODT “roadmap,” the surgeon decides whether surgery is the best option for managing the primary disease and with an oncologist for managing recurrent disease. The key here is that the right tools are selected to meet the individual needs of the patient’s case. If surgery is a “no-go,” the multidisciplinary team decides the best treatment options, as are the nature and timing of follow-up assessments. The decision to use an open or minimally invasive approach for surgery with curative intent determines the surgeon’s plan for obtaining the “intraoperative real-time assessment of EODT.” Again, a series of decisions must be made, including 1), if needed, which preferential locator to use; 2) what type of tracer, radionuclide vs. fluoroprobe, should label the PL; and 3) which intraoperative imaging cameras and detection probes to use. Real-time EODT assessment with concurrent use of an intraoperative gamma camera and detection probe allows the surgeon to image the surgical field, precisely excise DIT, and reimage the field before closure of the abdomen to ensure no unresected DIT remains, especially at the margins.

EODT for pathology may involve imaging the resected surgical specimen(s) and marking the site of DIT in the specimen before sending it to surgical pathology [9]. This approach correlates the pathologic assessment with the surgical findings, enhancing accurate staging and subsequent treatment decision-making. Does this “systems engineering” approach to surgical oncology work? We have successfully used this approach in patients with gastrinoma [10] and others with different solid tumors [6, 10, 11]. (See Figure 6 below.).

#### Colorectal Carcinoma as an Exemplar the Concept of EODT Assessment

Three different imaging modalities—anatomic, functional, or hybrid anatomic/functional—provide preoperative images. Based on morphologic imaging, current preoperative imaging techniques do not accurately map the EODT for CRC [15]. The National Comprehensive Cancer Network (NCCN) guidelines call for preoperative imaging using CT scans, with contrast, of the chest, abdomen, and pelvis [16]. Similarly, the American College of Radiology (ACR) recommendations include chest CT and magnetic resonance imaging (MRI) to assess rectal and colon cancer [17]. MRI is more commonly used to assess hepatic metastases and for staging rectal cancer. Variations in instrumentation, protocols, and level of experience of the reader play a critical role in the ability of CT and MRI imaging to recognize DIT, with a reported accuracy of approximately 70% for mapping lymph node metastases [18–20]. This problem is exemplified by Olsen and others [21], who found the inaccurate staging of 35% of primary CRC cases in their retrospective study of 3465 Danish patients, which included 36% of patients with regional metastases and 58% of patients with clinical stage I disease. Similarly, Reali et al. [22] reported in their study that preoperative staging in 948 CRC patients found correct T and N staging in only 19.68% of colon cancer patients compared to 53.85% of patients with rectal cancer. These discordant results, in part, are due to CT and MRI detection of lymph nodes, either benign or metastatic, are limited to those nodes 5 mm or larger and by the reproducibility of morphologic criteria [23]. However, metastases often occur in mesenteric lymph nodes below this 5 mm size detection level [24–27]. In a study of 6969 lymph nodes from CRC patients, Schrembs et al. [28] reported that the majority of lymph nodes identified fell between 1 and 6 mm in diameter, with 39% 3 mm or less in diameter, and 5.24 mm being the median size of positive lymph nodes as compared to 4.14 mm for negative lymph nodes. Current machine learning tools developed to improve this accuracy lack standardization in instrumentation, sample size, and algorithms needed to predict which preoperative CRC patients lack lymph node metastases [29, 30].

Unlike the anatomic information provided by CT and MRI imaging, molecular imaging provides functional information at the cellular and molecular levels. Molecular imaging requires a PL, a molecule (e.g., binding to a tumor-specific biomarker (e.g., protein, receptor) labeled with a radionuclide tracer. The tracer’s decay gives rise to photons imaged by SPECT or PET. Hybrid imaging, a combination of CT or MRI with either SPECT or PET, provides higher-resolution images than PET or SPECT alone. The image quality and spatial resolution of PET and PET/CT are improved. However, there are only a few clinical applications for SPECT and SPECT/CT in the preoperative imaging of solid tumors [31]. As evidenced by the recent Food and Drug Administration (FDA) approval of several new PET-based radiopharmaceuticals for neuroendocrine [32] and prostate carcinomas [33, 34], there is an increasing acceptance of molecular imaging in assessing EODT of solid tumors. Molecular imaging has led to many preclinical and early clinical studies of various biomarkers as possible targets for imaging in CRC and other cancers [35–39].

Armed with the preoperative image-derived roadmap, the surgeon must decide on the surgical approach. The success of a curative surgical procedure requires resection of all DIT. Based on an open surgical approach, our data indicates that if all gamma-positive tissue, regardless of pathology findings and staging, is removed, there is a significant overall survival advantage for patients with primary or recurrent CRC at 5 years or greater [40–42]. Most cases involved direct supervision by a select academic faculty specializing in surgical oncology or colorectal surgery. Today, minimally invasive surgery (MIS), either laparoscopic or robotic, is replacing the open procedure. MIS provides patients with a shorter hospital stay, reduced postoperative pain, improved bowel function return, reduced incidence of wound infections, and improved cosmesis [43]. The variability among surgeons’ technical experience with various open and minimally invasive procedures is well known [44, 45]. With MIS, the surgeon’s visual and tactile senses are often reduced. A potential drawback of laparoscopy is the inability to predict which patients require conversion to open surgery. Robotics reduce the problems of 2D visualization, maneuverability, and tremors by providing a stable camera, 3D magnification, and wrist-like dexterity. However, it comes at a much higher cost, longer operating room time, and reduced haptic control [46, 47]. The proposed EODT assessment approach will help reduce the learning curve and better predict patients with an open surgical procedure associated with MIS while adding auditory and visual information in real time regarding the extent and location of DIT. The surgical procedure will influence the subsequent selection of preferential locators, tracer labeling, and methods for detecting the tracer signal, especially in fluorescence-guided surgery cases.

### Examples of Preferential Locators

Cancer cells overexpress many biomarkers that are targets for PL in assessing EODT. However, there is no single cancer-specific target molecule. The ideal biomarker for PLs includes cellular and acellular components within the tumor’s extracellular matrix and molecules on the cell membranes of tumor cells. The latter includes overexpressed membrane glycoconjugates, proteins, receptors, transporters, and enzymes. In addition, intracellular molecules involved in altered cellular metabolic processes, e.g., cell proliferation or glucose metabolism, can serve as targets for PLs. It is important to note that the expression of these target molecules is heterogeneous among tumors, within a given tumor, among tumor cells, and between the primary and metastatic foci.

**FIGURE 2:**
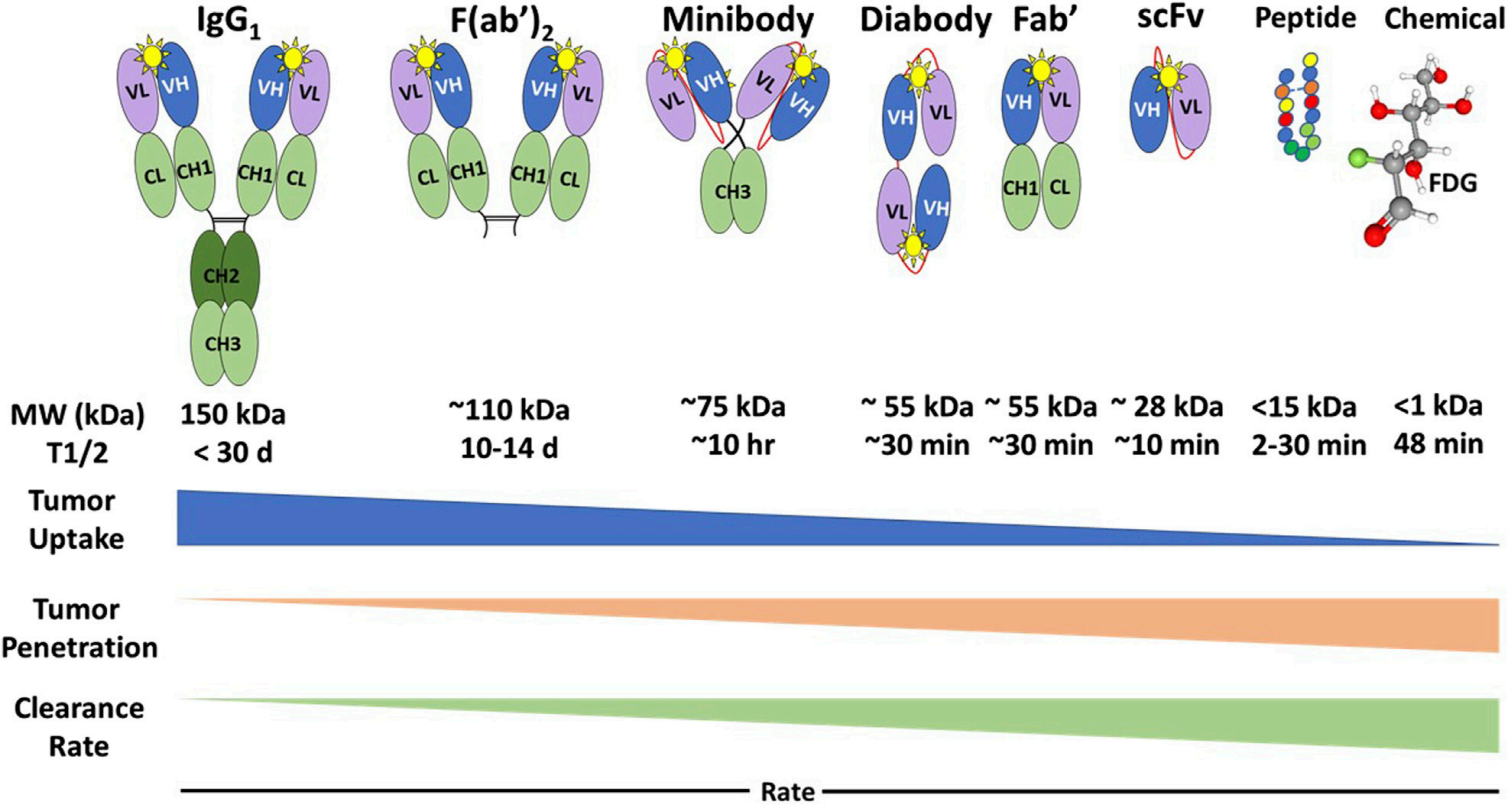
Types of Preferential Locators and Their Pharmacokinetics. FDG: ^18^F-fluorodeoxyglucose; MW, Molecular weight; kDa, kilodaltons; T_1/2_, half-life; Yellow star, antigenic epitope binding domain [12]. Generally, the PL’s tumor uptake rate is the inverse of its tumor penetration and clearance rate.

Regardless of the type of molecules, PLs for EODT assessment must exhibit high affinity (i.e., the ability for initial binding to the target molecule) and high avidity (i.e., the ability to remain bound to the target molecule over time) that allows the rapid clearance needed for a high tumor-to-background ratio (TBR) that optimizes imaging and detection of DIT. A PL’s molecular features—structure, size, mass, and charge—determine its pharmacokinetics and potential clinical applications (Figure 2).

The intact IgG monoclonal antibody (MoAb) has two identical heavy chains that contain a variable domain (VH) and three constant domains (CH1, CH2, and CH3), and two identical light chains containing a variable domain (VL) and a constant domain (CL). The IgG has two antigenic epitope binding domains (Figure 2, yellow star) formed by the two variable domains (VL and VH). Intact MoAbs have a slow plasma clearance, resulting in a 2-3-week half-life. Murine MoAbs (mMoAb) can induce unwanted human anti-mouse antibodies (HAMA) that can alter imaging results. Bioengineering of a chimeric MoAb with a murine or a human variable region and human constant regions minimizes immunogenicity while maintaining the affinity and avidity of the MoAb. The slow clearance allows for tumor uptake, penetration, and accumulation of the intact mMoAb but requires labeling with a radionuclide with a long half-life. This delay provides time for multiple imaging studies and optimization of surgical planning and intraoperative EODT assessment [46]. However, MoAb size requires hepatic rather than renal clearance, which may preclude the detection of liver metastases. In addition, the slow clearance and nonspecific binding by Fc receptors of intact mMoAbs can lead to decreased TBRs [48].

Enzymatic digestion removes the constant regions from the mMoAb to yield smaller Fab’ and F (ab’)2 fragments that exhibit rapid tumor penetration and increased renal clearance without altering antigen binding. The bioengineered single-chain fragment variable (scFv) fragments, containing a variable light chain (VL) and a variable heavy (VH) chain, require a linker molecule for stability and are easier to produce [49]. The scFv can give rise to multivalent diabody, triabody, tetrabody, and minibody (scFv-CH3) fragments with the same avidity, affinity, and optimal pharmacokinetics (Figure 2). There are even smaller, 15 kDa, single domain VH chains or nanobodies, small peptides, and chemicals that are potential PLs for molecular imaging. These bioengineered PLs retain the high affinity and avidity of the intact mMoAb while providing improved renal clearance and tumor penetration. The result is a better TBR than the parent mMoAb and, thus, a better EODT image [48]. In cases where there is a need to increase the serum half-life of smaller PLs to optimize tumor uptake and reduce renal clearance, this is accomplished by adding discrete sizes of polyethylene glycol to the PL [12, 50].

Small molecules such as the glucose analog 18F-fluorodeoxyglucose (FDG) and fibroblast-activating protein inhibitor (FAPI) have a biological half-life measured in minutes, facilitating their use for preoperative molecular imaging (Figure 2). PET/CT, using the radionuclide-labeled glucose analog 18F-FDG as the PL, is the current “go-to” for hybrid molecular imaging for non-small cell lung cancer, head and neck cancers, and lymphomas. ^18^F-FDG-PET/CT is helpful in selected CRC cases for identifying metastases; however, its low specificity minimizes its use in preoperative imaging [51, 52]. The increased uptake of 18F-FDG by inflamed tissue, wound healing, and infections results in false positive images, whereas slow-growing tumors exhibit no increase in ^18^F-FDG uptake [53].

Although not FDA-approved, PET and PET/CT using radionuclide-labeled FAPI show promise as a replacement for ^18^F-FDG in preoperative imaging of CRC. Upregulation of fibroblast-activating protein (FAP) expression by cancer-associated fibroblasts occurs in 90% of primary and metastatic carcinomas [54]. In the case of CRC, the improved TBR of ^68^Ga-FAPI PET/CT over 18F-FDG-PET/CT leads to improved identification of primary and recurrent disease and in the detection of lymph node metastases [55]. Similar head-to-head studies of other tumors demonstrate that EODT using FAPI is equal to or superior to FDG in many tumors, including CRC. However, neither FDG nor FAPI binding is cancer-specific. False positive imaging occurs in wound healing and various diseases characterized by fibrosis, e.g., cirrhosis, healing myocardial infarcts, and autoimmune diseases. False negative imaging is associated with tumors lacking accumulation of cancer-associated fibroblast, e.g., leukemia, lymphomas, and multiple myeloma [54].

Even though there are multiple FDA-approved MoAbs for therapy, there is only a limited number for imaging. Many previously approved imaging products are no longer on the market in the United States [56]. Many of the early SPECT-related PLs were taken off the market, partly due to the increasing use of ^18^F-FDG-PET and ^18^F-FDG-PET/CT and issues with image quality (Table 1). As noted earlier, developing new PLs for neuroendocrine and prostatic carcinomas is reestablishing the central role of preoperative molecular imaging in providing an accurate EODT road map [32, 57]. Based on decades of clinical rather than preclinical results, we propose that the same success can be attained for EODT assessment in CRC using newer versions of PLs specific for the tumor-associated glycoprotein-72 antigen (TAG-72) and for the carcinoembryonic antigen (CEA), a member of the carcinoembryonic antigen cell adhesion family of molecules (aka CEACAM5). The nonspecific tumor-specific expression of TAG-72 and CEA is associated with 80%–95% of CRC primary and recurrent tumors. Both are large membrane-bound glycoproteins found in a diverse group of carcinomas and a few non-malignant conditions. However, except for secretory endometrium with TAG-72, it shows little or no expression in normal tissue [8, 58].

**TABLE 1:** FDA-Approved Preferential Locators for Molecular Imaging and Fluorescence-Guided Surgery. * No longer marketed in the U.S.; FDG: ^18^F-fluorodeoxyglucose; DCFPyl: 2-(3-{1-carboxy-5-[(6-^18^F-fluoro-pyridine-3-carbonyl)-amino]-pentyl}-ureido)-pentanedioic acid; PSMA: prostate specific membrane antigen; NET: neuroendocrine carcinoma; TAG-72: tumor associated glycoprotein-72; NIR: near infrared; FGS: fluorescence guided surgery.

Preferential locator (FDA approval Date)	Product Name	Tumor	Target	Tracer	Application
Small Molecules					
Fluorodeoxyglucose (8/1999)	FDG	Proliferating Cancers	Functional	^18^F	PET
Pentetreotide (11/1998)	Octreoscan	NET	Somatostatin Receptor	^111^In	SPECT
1,4,7,10-tetraazacyclodo-decane-1,4,7,10-tetraacertic acid (DOTA)-octreotate (6/2016)	DOTATATE	NET	Somatostatin Receptor	^68^Ga	PET
PSMA-11 December 2020	Illuccix	Prostate Cancer	PSMA	^68^Ga	PET
DCFPyl (5/2021)	Pylarify	Prostate Cancer	PSMA	^18^F	PET
^18^F-rh-PSMA-7.3 (6/2023)	Posluma	Prostate Cancer	PSMA	^18^F	PET
Pafolacianine (11/21)	Cytalux	Lung and Ovarian Cancers	Folate Receptor	NIR	FGS
**Antibodies**					
B72.3* Murine IgG (1992)	Oncoscint	Colorectal Cancer	TAG-72	^111^In	SPECT
NR-LU-10* Murine Fab (1996)	Verluma	Small Cell Lung Cancer	CEA	^99m^Tc	SPECT
NP-4 Fab* (1999)	Arcitumomab	Colorectal Carcinoma	CEA	^99m^Tc	SPECT
7E11-C5.3* Murine IgG (1996)	ProstaScint	Prostate Cancer	PSMA	^111^In	SPECT

TAG-72 is a mucin-like glycoprotein biomarker for CRC and other adenocarcinomas. TAG-72 has a single protein core with extensive O-linked glycosylation that accounts for 80% of its molecular weight of 1,000 kDa [59]. The function of TAG-72 is unknown but may be capable of inhibiting the immature dendritic cells in the tumor and in tumor-draining lymph nodes [14]. Immunohistochemical (IHC) staining demonstrates TAG-72 in cytoplasmic secretory vacuoles on the extracellular matrix’s plasma membranes, luminal secretions, and mucin lakes [14].

The last 30 years saw multiple generations of mMoAbs developed as PLs for TAG-72 (Table 2). Over 1,000 patients have safely received one of several generations of mMoAbs to TAG-72 in clinical studies of various carcinomas (Table 2). [41] The first-generation anti-TAG-72 mMoAb B72.3 received FDA approval for SPECT imaging. Numerous single-institution and multicenter clinical trials of primary and recurrent CRC for radioimmunoguided surgery (RIGS) used B72.3 [73–77]. CC49 is the second generation mMoAbs to TAG-72 and has an improved affinity and avidity for TAG-72 compared to B72.3 [61, 78–80]. IHC staining with CC49 demonstrates TAG-72 in 86% of primary CRC tumors and 96% of recurrent tumors. CC49 provides the starting material antibody fragments and bioengineered derivatives of scFv, which are in preclinical and early phase I/II studies (Table 2). The use of mMoAbs to TAG-72 has been directly correlated to patient overall survival in the setting of RIGS or what we now refer to as Antigen-Directed Cancer Surgery (ADCS), but not in the context of preoperative PET/CT imaging [42].

**TABLE 2:** Generations of monoclonal antibodies to TAG-72: Their source and applications.

Anti-TAG-72 MoAb	Generation	Type	Applications (patients injected)	References
B72.3	1st	Murine IgG	SPECT Phase I and II RIGS (N = 805)	[41, 60]
CC49	2nd	Murine IgG	Phase I and II RIGS/ADCS SPECT—Pilot (N = 459)	[41, 61, 62]
CC49	2nd	Fab’	Preclinical MicroPET/CT	[62]
CC83	2nd	Murine IgG	Pilot—RIGS/ADCS (N = 17)	[63]
HuCC49	3rd	Bioengineered IgG	Preclinical Fluorescent Imaging	[64]
HuCC49∆(CH)2	3rd	Bioengineered Chimeric IgG From CC49	Pilot—RIGS/ADCS (N = 20)	[65–68]
3E8	4th	Bioengineered Humanized IgG From CC49	Preclinical Pharmacokinetics	[69]
AVP04-7-PEG	5th	Bioengineered Humanized scFv and Diabody From CC49	Pilot - PET (N = 6)	[50, 70, 71]
3E8.scFv.Cys	5th	Bioengineered Humanized scFv From 3E8	Preclinical FL Imaging	[72]
ENL-210	5th	Bioengineered Humanized scFv—diabody -tetrabody From 3E8	Preclinical Imaging	[49]

Over the last 40 years, numerous anti-CEA mMoAbs were developed and used for serum detection, IHC staining, preoperative and intraoperative imaging, radiation therapy, and immunotherapeutics. The mMoAbs to CEA bind to one of the nine domains (A1, B1, A2, B2, A3, B3, and N) that form the 79 kDa core protein [81–86]. The last decade has steadily progressed from preclinical to the current phase III clinical trial using mMoAb SGM-101, a chimeric IgG in the A2 domain of CEA core protein. Later studies focus on FSG, which requires intraoperative imaging resources [87]. The results of a Phase II trial demonstrated tumor involvement of the peritoneum, superficial lymph nodes, and surgical margins that improved real-time surgical decisions. In contrast to fluorescence imaging, a recent study reported using a humanized chimeric mMoAb recognizing the A3 domain of CEA for preoperative PET/CT imaging of 20 patients with either gastric, colorectal, medullary thyroid, or neuroendocrine carcinoma [88]. Although the mMoAb had a high affinity for CEA, the authors concluded that the mMoAb is optimal for imaging patients with locally advanced rectal carcinoma. This last study highlights the impact of PLs on the endpoints selected for clinical studies. However, numerous ongoing clinical trials use various PLs for imaging and therapy of solid tumors [56, 89].

#### Tracer Molecules

**FIGURE 3:**
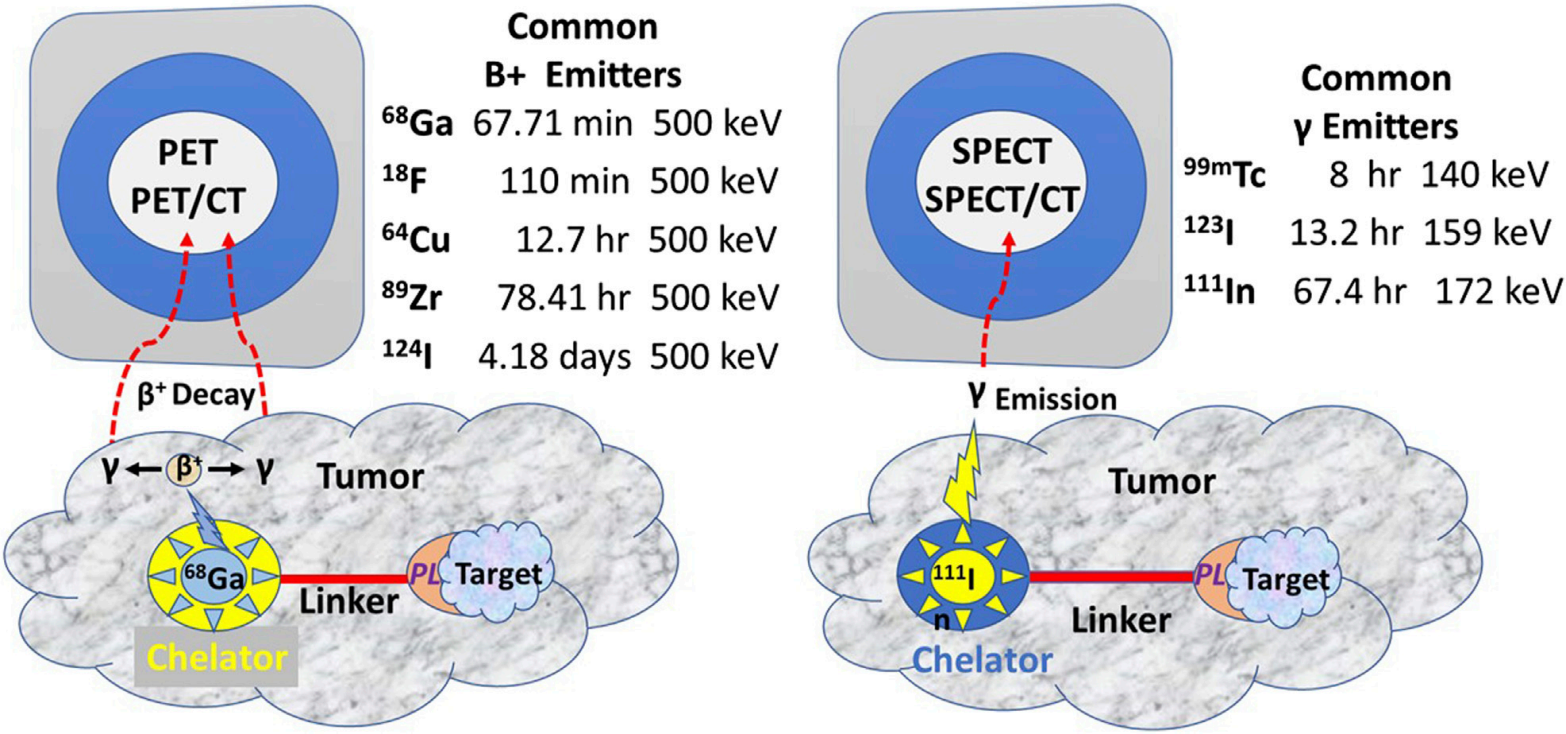
Commonly Used Radionuclides for PET and SPECT Imaging (After [13] PET, Positron emission tomography; SPECT, Single-photon emission computed tomography; CT, Computerized tomography; PL, Preferential locator.

The tracer molecule (radionuclide or fluorophore) linked to the PL bound to the target molecule produces the signal (Figure 3). The molecular relationships among the components of the radiopharmaceuticals—the PL, the tracer, the bifunctional chelator, and the linker—are critical to optimizing the TBR needed for an accurate EODT assessment. Optimizing results begins by closely matching the linked radionuclide’s half-life with the PL’s biological half-life.

In contrast to iodine radionuclides, radiometals used for PET and SPECT require a chelator to form a stable complex with the metal ion to prevent metal loss after the intravenous injection and form a bifunctional complex by linking to the PL for injection. Chelators reduce the harsh conditions for labeling a PL with 18-fluorine. A linker molecule joins the chelated radionuclide to the PL (Figure 3). Radionuclide tracers for PET imaging undergo B+ decay, yielding two high energy gamma (γ) rays 180° apart. However, the improved sensitivity of the PET, and thus the better EODT preoperative roadmap, is more a function of the instrumentation rather than the number of gamma rays emitted. High-energy radionuclides have far greater tissue penetrance, allowing for imaging and detection at a greater distance from the source and for detecting small sources.

The TBR is the critical variable allowing intraoperative detection and imaging of small (<5 mm diameter) gamma-positive tissue. In our experience, this requires the physical half-life of the radionuclide (referred to as radionuclide decay T1/2) to be at least five times that of the biological half-life due to clearance from normal tissue [90, 91]. PET and PET/CT use a high-energy radionuclide-bound PL for preoperative molecular imaging. In the initial EODT assessment for CRC cases, we propose using 124I to label the PL. This high-energy radionuclide has a physical half-life of 4.2 days and a biological half-life of approximately 61 days, which allows for imaging over several days and optimizing the TBR for a more accurate EODT roadmap. The intraoperative EODT assessment for CRC currently calls for using a low-energy radionuclide. We recommend 123I, bound to the same PL used for the “roadmap” for intraoperative imaging and detection. 123I has a physical half-life of 13.2 h compared to a biological half-life of 120–138 days if unbound. The ease of labeling a PL with 124I for PET imaging and 123I for intraoperative EODT assessment should not alter the affinity or the avidity of the PL. To further optimize the TBR while maintaining the avidity and affinity of the PL, we propose an EODT assessment of CRC using the chimeric anti-TAG-72 MoAb, HuCC49∆CH2, that is currently available. HuCC49∆CH2, with its complementarity determining region (CDR)-grafted humanized domain-deleted CC49 MoAb, was safely administered to 20 patients with recurrent CRC [91]. Pharmacokinetic studies indicate that its physical half-life is 1.34 days, its biologic half-life is 12.8 days, and renal excretion accounted for over 66% of the clearance [66].

**FIGURE 4:**
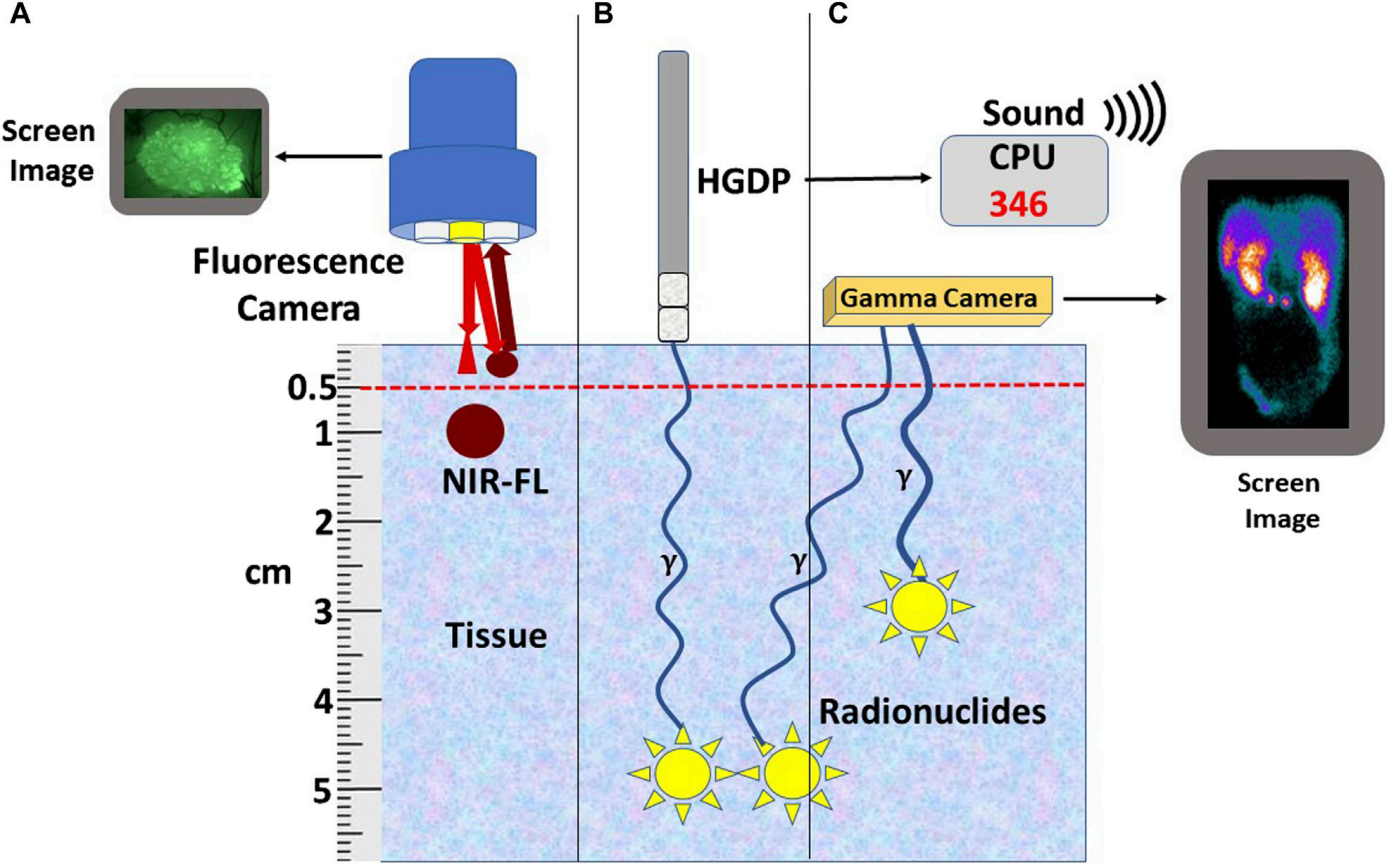
Relationship of Tracer to Depth-of-Signal Detection. **(A)** Currently available fluorescent cameras do not detect near-infrared fluorescence-labeled preferential locator (PL) (NIR-FL) signals deeper than 5 mm of tissue; however, similar NIR-FL labeled tumors within 5 mm of the surface can be detected. **(B)** Radionuclide-labeled PLs (yellow star) bound tumors located deep in the tissue can be detected using a handheld gamma-detecting probe (HGDP). The 3-sigma counts over background are expressed numerically and audibly on the central processing unit (CPU). **(C)** A gamma (γ) camera detects the radionuclide-labeled PL (Yellow stars) converted into a 2D image. Bringing the HGDP and the γ-camera close to the signal’s source negates the short depth-of-penetrance of low-energy radionuclides.

Preclinical and clinical research into fluorophore-guided surgery is expanding [6, 92–103]. An international, multicenter, phase III clinical trial for intraoperative EODT assessment in primary and recurrent CRC cases is underway [104]. The study employs a near-infrared (NIR) fluorophore (carbocyanine dye (BM105) labeled anti-CEA mMoAb (SGM-101) for real-time intraoperative assessment for positive surgical margins. The problem of positive margins varies with the tumor type [9] and occurs in over 15% of all resected cancers [105]. This procedure also has the advantage of real-time assessment for occult carcinomatosis [106]. However, the inability of NIR light to penetrate beyond 5 mm (Figure 4) minimizes its ability to detect tumor-involved regional and extra-regional lymph nodes seen on preoperative images. Current FDA clinical approval of only two NIR dyes in the 700–900 nm range—indocyanine green and methylene blue—must be expanded to include other NIR dyes whose emission wavelength has deeper tissue penetration and a better TBR. In addition, the future holds great promise for combining both types of tracers on the same PL [102, 107, 108].

#### Intraoperative Imaging and Detection of the Tracer Signal

There is ongoing research and development of medical devices for intraoperative imaging and detecting radionuclides and NIR fluorophores. Further proof of the proposed concept for intraoperative EODT assessment is well underway with the FDA approval of 68GA-PSMA-11 for primary and recurrent prostate cancer [33] and with the current phase III trial with the NIR fluoroprobe labeled anti-CEA SGM-101 [92], as well as the fluorescence-guided ovarian and lung cancer surgery using Pafolacianine [100, 109].

**FIGURE 5:**
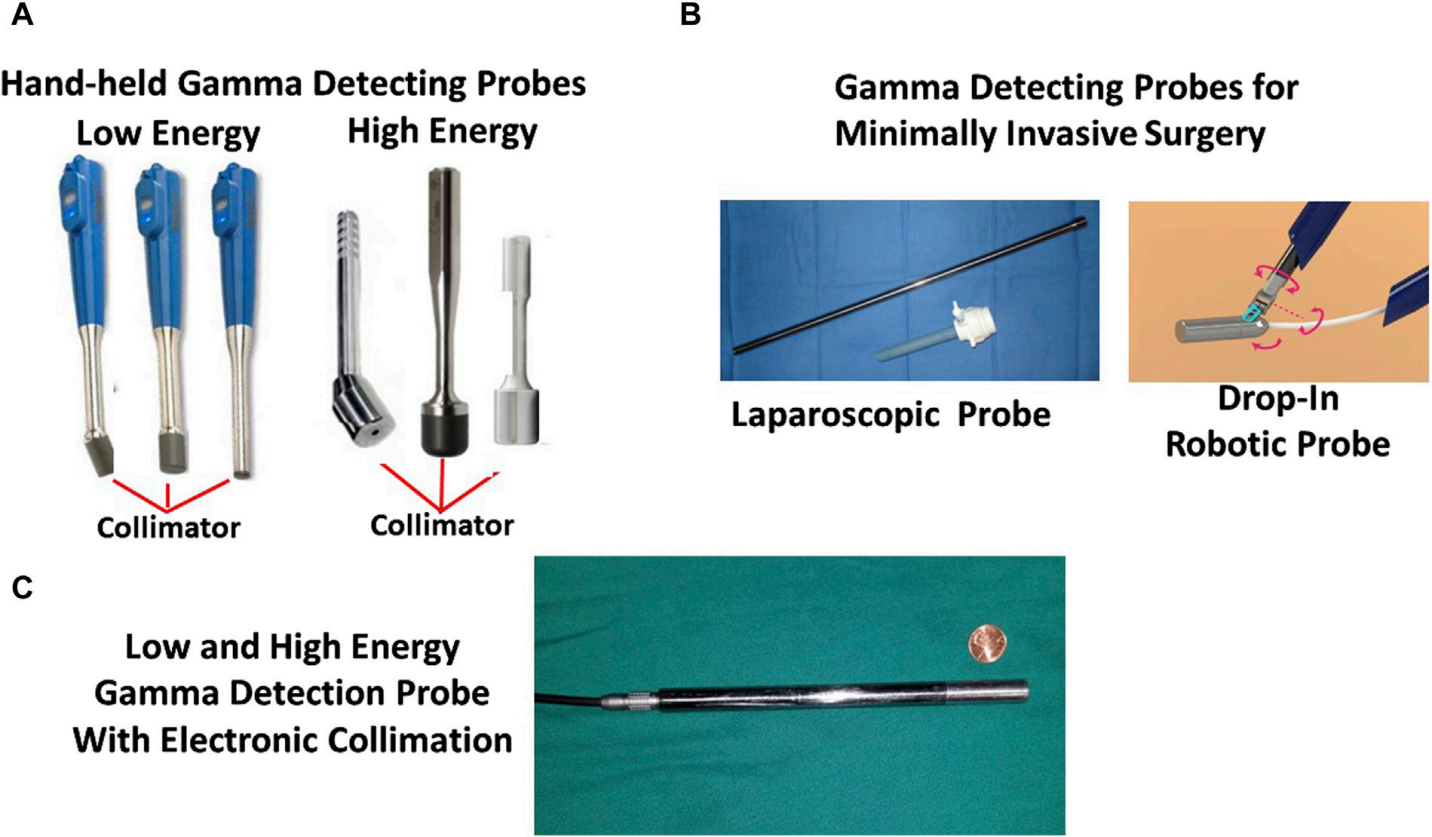
Intraoperative Gamma Detection Probes. **(A)** Three commercially available Bluetooth-enabled low-energy handheld gamma-detecting probes (HGDPs) and a laparoscopic gamma-detecting probe and trocar. **(B)** Three commercially available high-energy HGDPs with prominent collimators and a drawing of a proposed drop-in gamma detecting probe for robotic minimally invasive surgery (MIS). **(C)** Newly developed 2nd generation gamma detection probe for detecting high- and low-energy radionuclides and with electronic collimation.

The type of equipment is defined by the type of surgery—open vs. minimally invasive—and by the depth of the tracer signal within the tissue (Figure 4). By themselves, PET and SPECT molecular imaging have limitations in detecting DIT smaller than 5 mm in the greatest dimension. The primary reasons are that the detectors are several centimeters away from the source and have a relatively low sensitivity to detect low-energy emitted gamma rays [103]. Thus, to find gamma-positive tissue smaller than 5 mm requires a detector to be close to the source. The introduction of the sentinel lymph node biopsy as the standard of care for several cancers and the ability of a handheld gamma-detecting probe (HGDP) in direct contact with the tissue led to the development of a wide range of probes and control units (Figure 5). Many of the current low-energy gamma-detecting probes are Bluetooth-enabled. HGDPs for high-energy radionuclide detection are not ergonomic due to additional shielding that increases probe weight, restricting them to open procedure use. The central processing unit, or console, provides a digital count rate and audio output that may vary its pitch based on the count rate. The control unit uses a three-sigma criterion—he baseline count plus three times the square root of the baseline or greater to provide a positive count [90, 110]. We designed the next-generation of gamma probes (e.g., handheld and MIS compatible) capable of detecting low and high-energy radionuclides using electronic collimation (U.S. Patents Nos. 11,467,295 and 11,562,454) that improves probe sensitivity by eliminating the need for physical collimation [111]. Optimal intraoperative EODT assessment occurs with the gamma-detecting probe, capable of being placed close to the source of the signal, used in conjunction with a portable small or portable large field-of-view gamma camera. Reviewed by [112] In addition to providing the required field-of-view, resolution, and sensitivity, the surgeon needs real-time monitoring and no delay in acquisition and display. The handheld cameras have the same disadvantage as the gamma probes in that they depend on the ability of the user to position the probe toward the source correctly. We have used a portable SPECT gamma camera (Ergo) in conjunction with HGDPs for six cases with recurrent gastrinoma that demonstrated 100% pathologic correlation with the imaging findings [10]. Similar results occur in SPECT imaging of 99mTc-Sestamibi for parathyroid adenomas [113].

Lauwerends et al. [97] defined fluorescent-guided surgery (FGS) as “an optical imaging method that provides real-time guidance for delineation of [tumors] during surgery, with higher sensitivity than direct visual inspection and palpation.” As with gamma probes and cameras, near-infrared (NIR) fluorescent imaging systems provide both ergonomic and real-time information to ensure adoption by surgeons. In the CRC setting, these systems assess surgical margins, lymph nodes, and peritoneal surfaces for occult-tumor in EODT assessment of CRC [87, 114]. These include systems for open, laparoscopic, and robotic FGS procedures. Commercial systems for FGS vary in their features. Reviewed in [110] As indocyanine green (ICG) is the only FDA-approved NIR fluorescent dye useful for FGS studies, various excitation light sources—laser diodes and light-emitting diodes—include NIR bandwidth. Each system varies according to its working distance, field of view, image contrast, and quality. Charge-coupled device cameras provide high-resolution and cost-efficient means of capturing the emitted photons and provide qualitative rather than quantitative EODT images. Ongoing development of new sensors indicates that the improved depth of fluorescent signal detection is greater than the accepted 5 mm value.

### Proofs of the Concept

**FIGURE 6:**
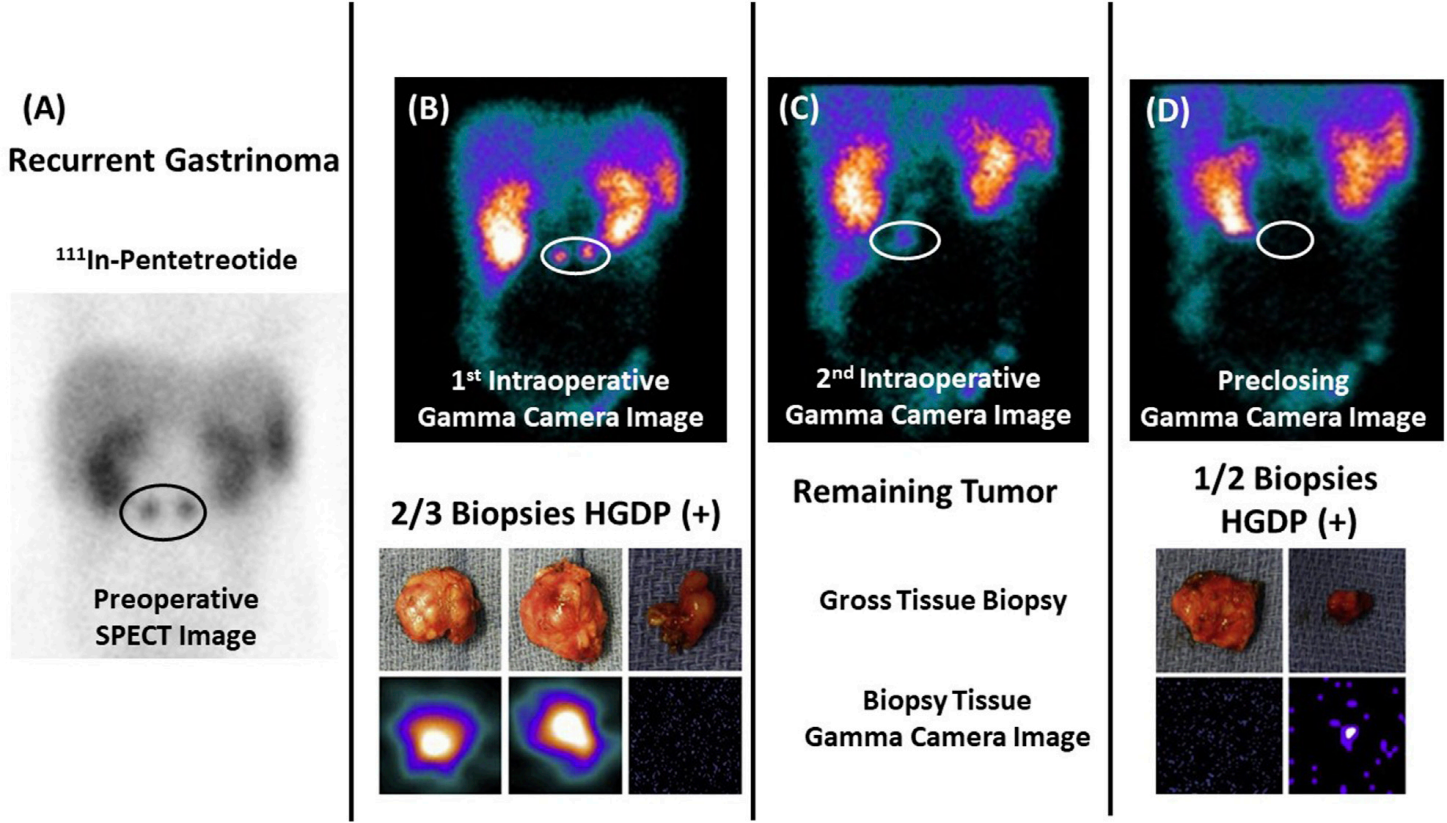
Assessing Extent of Diseased Tissue (DIT) in a Gastrinoma Patient **(A)** Two abdominal masses consistent with recurrent gastrinoma are seen on a preoperative ^111^In-pentetreotide SPECT scan in a 17-year-old patient with elevated serum gastrin levels. **(B)** Images from a portable intraoperative, large field-of-view gamma camera were used to locate the DIT (upper image—white oval) and the three resected specimens (lower image)—two (left and middle images) of which were diagnosed as “positive” by the nuclear medicine physician. **(C)** The nuclear medicine physician noted the remaining DIT upon reimaging the surgical field (white oval). **(D)** The handheld gamma-detecting probe (HGDP) detected the obscure DIT inside the duodenum, and two specimens were removed and reimaged (lower image) for confirmation. The preclosing image showed no remaining DIT (upper image). In this case, the pathologist knew exactly the tissue specimens requiring careful examination! After [10, 14].

**FIGURE 7:**
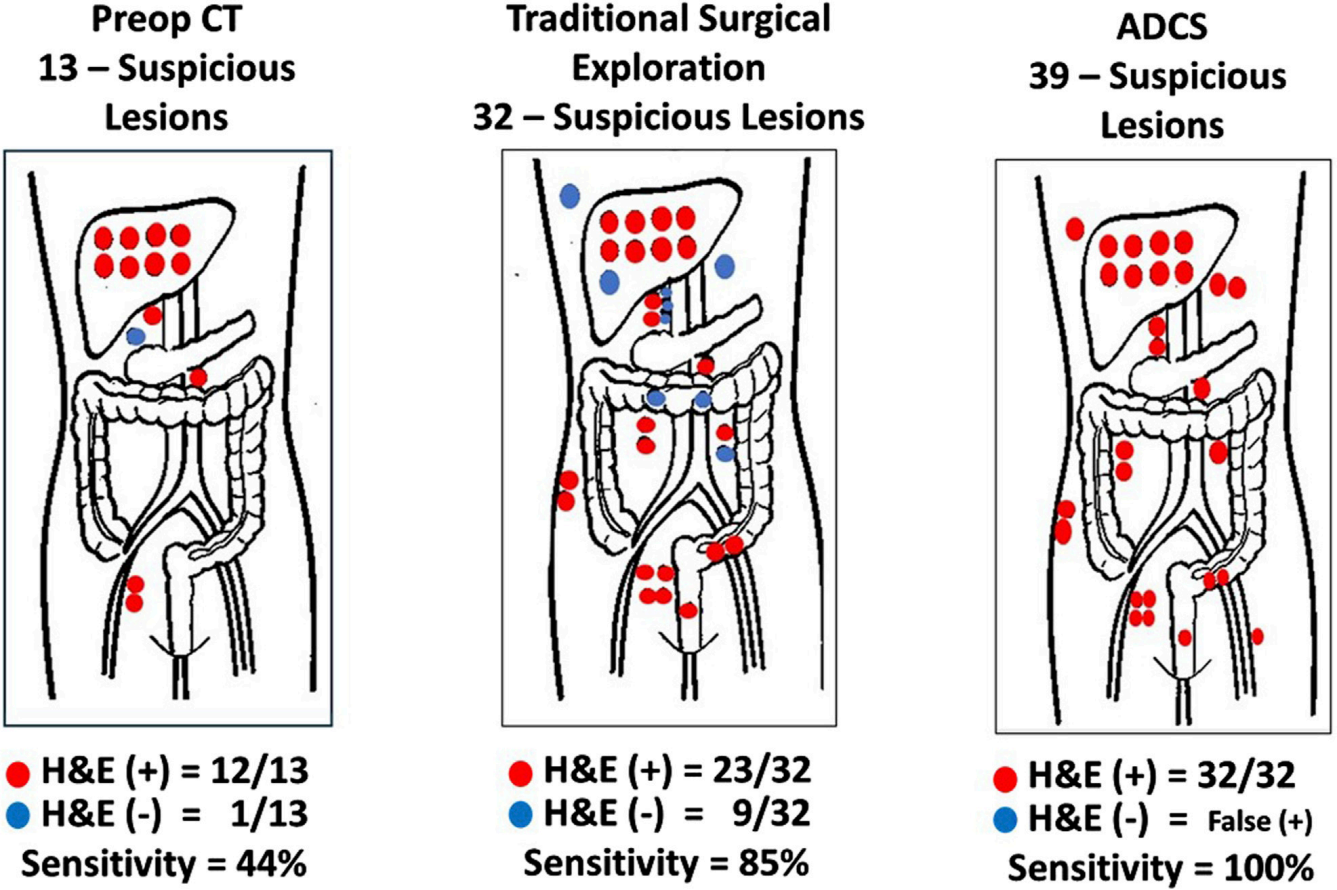
Sensitivity and Positive Predictive Value of Methods for Detecting Disease-Involved Tissue (DIT). Red dots hematoxylin and eosin (H&E) (+) biopsy; Blue dots H&E (−) biopsy; ADCS: Antigen-directed Cancer Surgery; CT: Computerized tomography; Traditional Surgery: surgeon’s visual and manual inspection of surgical field. (After 74).

**FIGURE 8:**
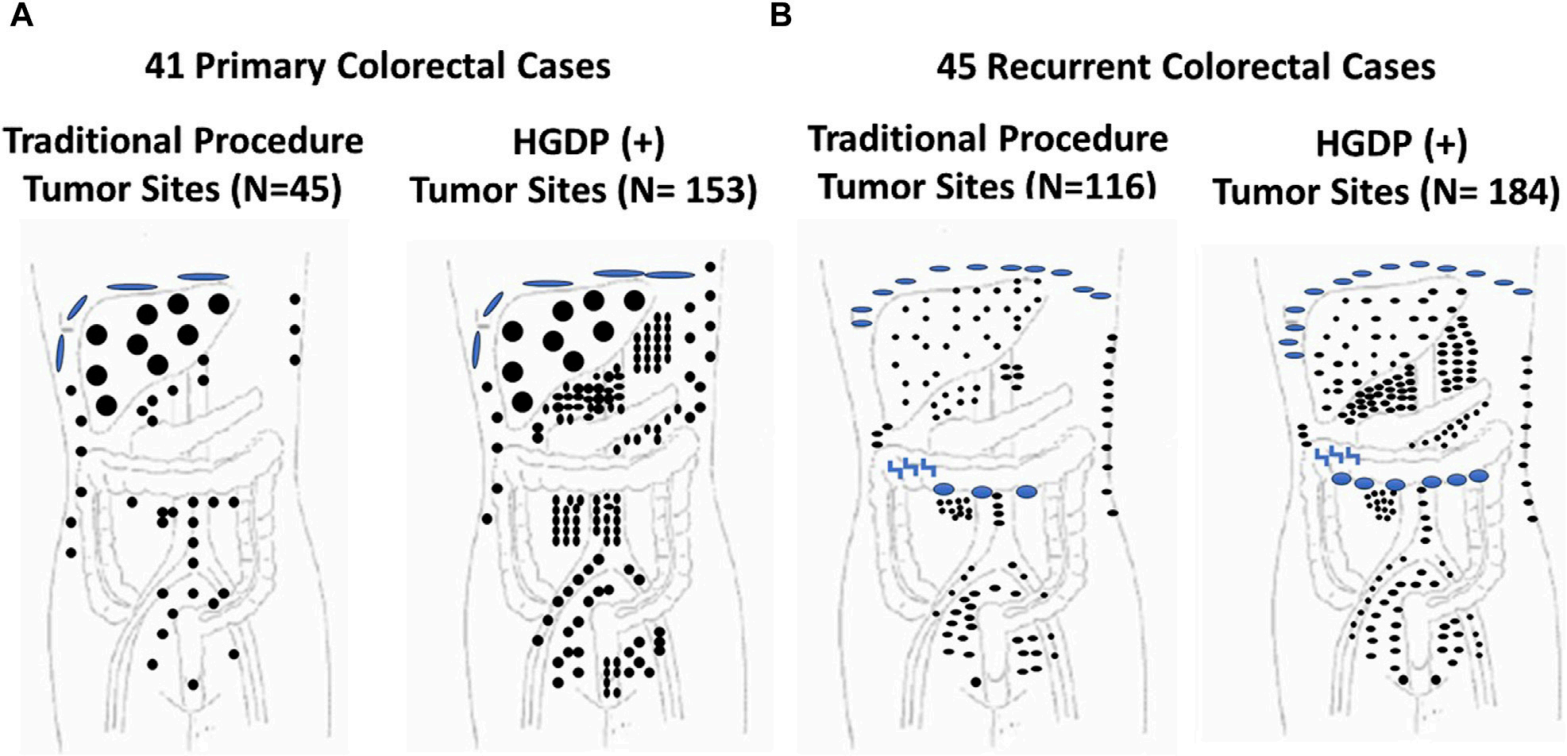
Traditional Exploration Using Visual Inspection and Palpation Techniques vs. Handheld Gamma-detecting Probe (HGDP) in Assessing Extent of Diseased Tissue (EODT) in Extra-regional Locations in Primary and Recurrent Colorectal Carcinoma (CRC) **(A)** Primary CRC Cases: HGDP (+) disease involved tissue (DIT): subdiaphragmatic implants (large blue disks), liver (large black circles), abdominal wall (three upper right small black dots), and potential lymph nodes (black dots). **(B)** Recurrent CRC Cases: HGDP (+) DIT: subdiaphragmatic implants (small blue disks), carcinomatosis (large blue ovals), serosal implants (blue inverted z), abdominal wall (right vertical small black dots) and potential metastatic disease (small black dots). After [115]

**FIGURE 9:**
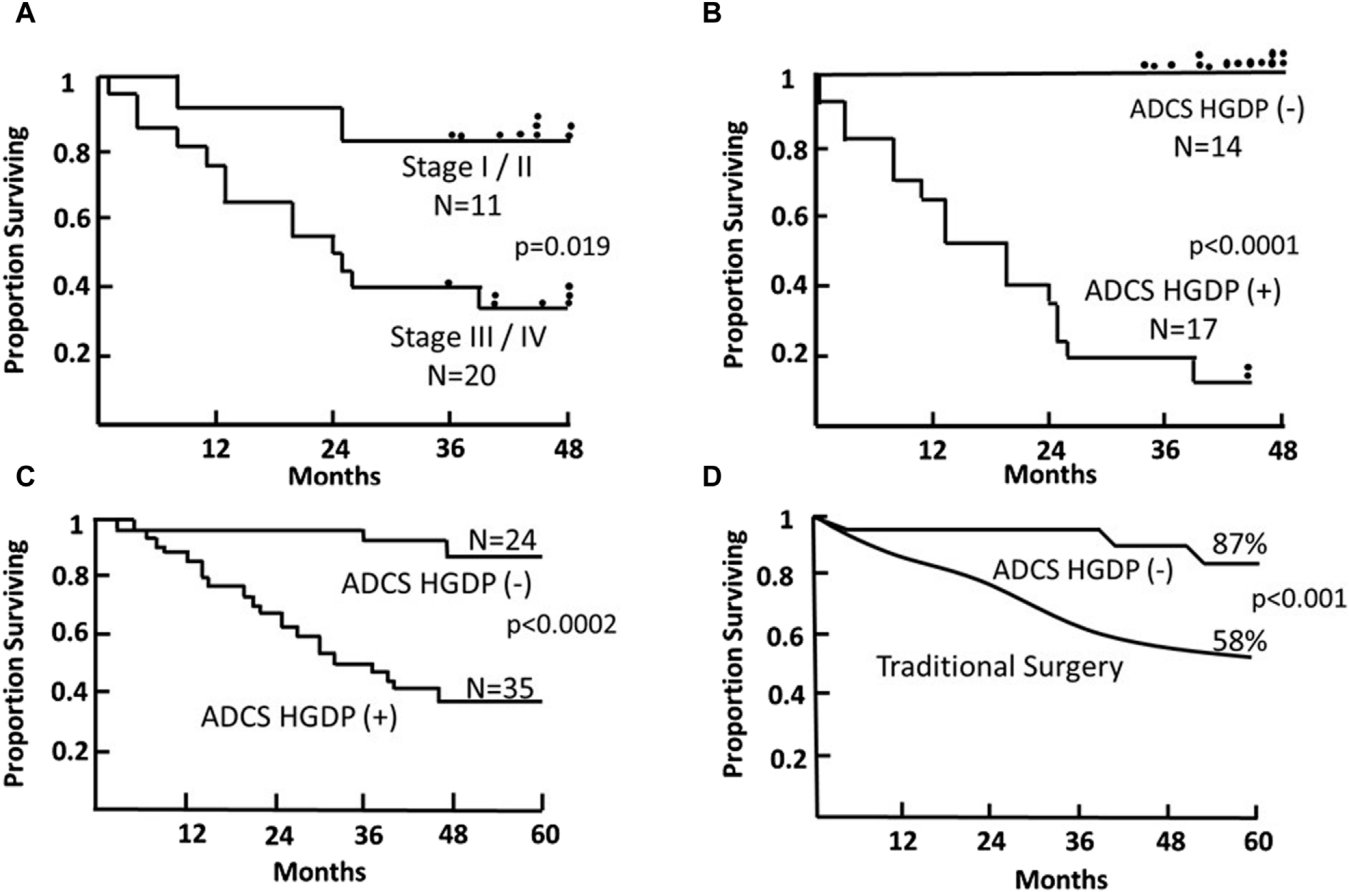
Survival of Patients with Primary Colorectal Carcinoma (CRC) Following TAG-72 Antigen-Directed Cancer Surgery (ADCS). Panels **(A,B)** compare the 4-year survival of 31 patients based on pStage criteria **(A)** vs. resection of all disease-involved tissue (DIT) **(B)** following injection with ^125^I-B72.3 and ADCS for assessing the extent of disease-involved tissue (EODT) with the presence or absence of handheld gamma-detecting probe (HGDP) (+) tissue remaining at the end of surgery [116]. **(C)** Five-year survival of 59, pStage I-III, primary CRC patients who received 125I-CC49 and underwent ADCS for EODT assessment with the presence or absence of HGDP (+) tissue remaining at the end of surgery [117]. **(D)** Comparison of the 5-year survival of the same 24 ADCS HGDP (−) patients in **(C)** vs. a pStage, gender, and age-matched control group of primary CRC patients who underwent traditional surgery at the same institution. After [118]

**FIGURE 10:**
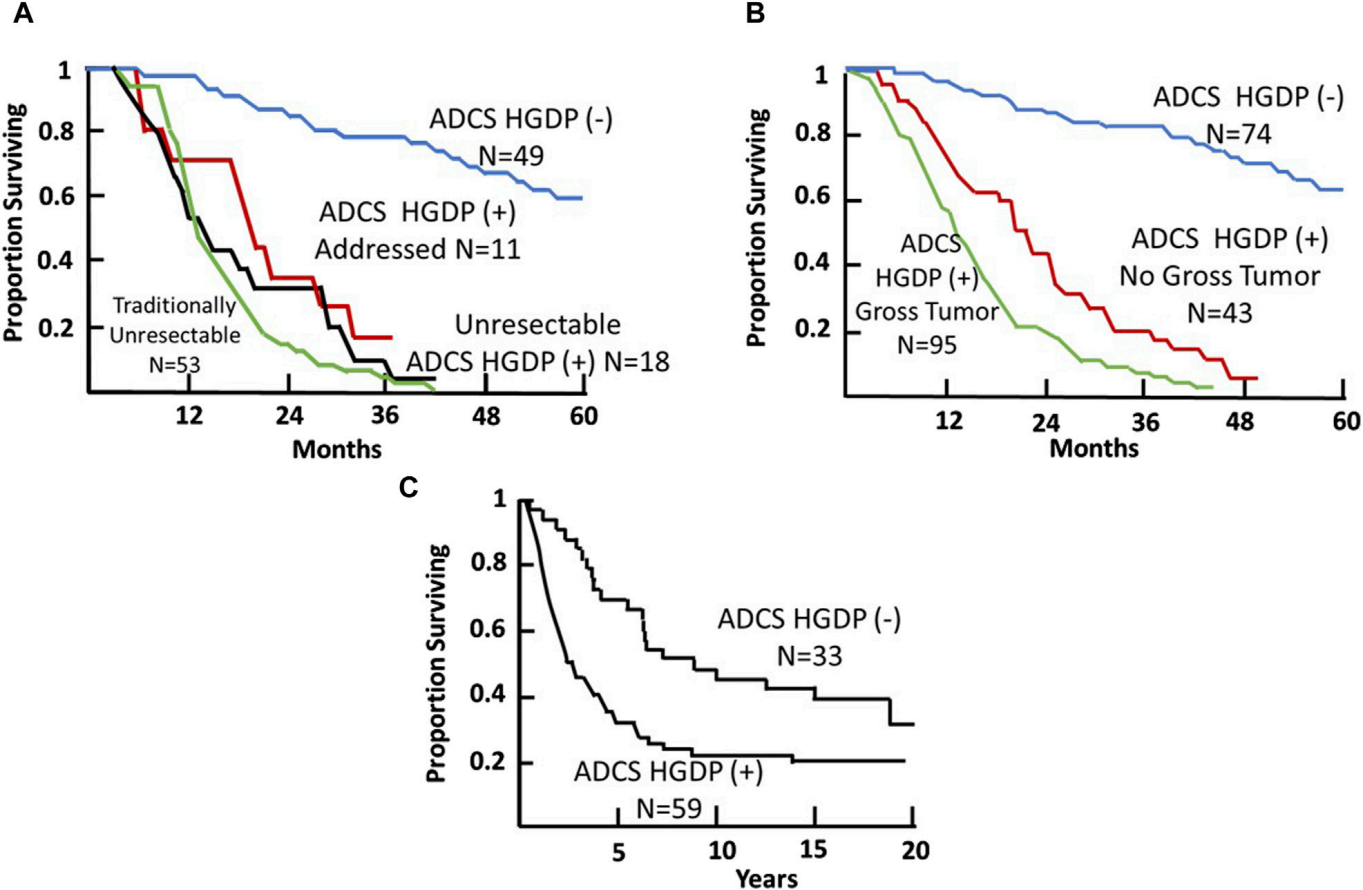
Colorectal Carcinoma (CRC) Patient Survival Following TAG-72 Antigen-Directed Cancer Surgery (ADCS). **(A)** Five-year survival recurrent CRC ADCS cases injected with ^125^I-B72.3 or ^125^I-CC49 (N = 131) [119]. **(B)** Five-year survival of primary and recurrent CRC ADCS cases injected with ^125^I-labeled B72.3, CC49, or CC83 (N = 212) [38]. **(C)** Fifteen-year survival of primary CRC ADCS cases injected with ^125^I-CC49 (N = 92). (After 39).

Our clinical studies demonstrate that the proposed approach (Figure 1) works. By providing the surgeon with an accurate assessment of the EODT intraoperatively (Figures 6–8), the surgeon is more likely to achieve a complete R0 resection, improving patient survival (Figures 9, 10).

#### An Accurate Assessment of EODT Provides Added Value to the Surgeon and the Patient

In the case of neuroendocrine carcinomas, Figure 6, we used the same radionuclide labeled PL (^111^In-pentetreotide) for the preoperative and intraoperative imaging in conjunction with an HGDP for intraoperative detection of the gastrin-secreting tumor. Figure 6 demonstrates our proposed approach’s ability to assess the EODT from the initial preoperative imaging to intraoperative detection and imaging prior to closing [10].

Pathologist knew exactly the tissue specimens requiring careful examination! (After 10 and 58).

#### Increasing Detection DIT in Colorectal Carcinoma

In the case of primary and recurrent CRC cases, inadequate preoperative imaging hampered our earlier clinical studies. A careful and detailed exploration of the abdomen and pelvis using an HGDP partially overcame the lack of an intraoperative gamma camera. Although this approach had a significant impact on the surgical procedure itself, having a “large field” gamma camera intraoperatively to demonstrate diseased tissues would have impacted patient morbidity and mortality. This conclusion is based on the fact that, under the term radioimmunoguided surgery (RIGS), we repeatedly demonstrated the truism “that it is what is left behind kills the patient, not what the surgeon takes out” and that traditional surgical procedures leave a lot of DIT behind (Figures 7–10). Achieving an R0 resection is the most important variable associated with recurrence and long-term survival; however, it may not be possible due to the extent and location of DIT.

The first step in assessing the EODT is locating DIT as defined by the binding of the PL. Two early studies demonstrate this premise. Figure 7 demonstrates the accuracy of ADCS (RIGS), using ^125^I-CC83, a 2nd generation mMoAb to TAG-72, for DIT in 17 patients with CRC [63]. This small study demonstrated that the sensitivity of the preoperative CT scan was only 44%, a finding that tends to support the conclusion by Brouwer et al. [120] that the “accuracy of clinical lymph node staging in colorectal cancer patients is about as accurate as flipping a coin.” It also demonstrated that while the more subjective, traditional surgical visual and palpation performed better than the CT scan in assessing EODT, the sensitivity of ADCS to detect tumors was 100%, but the presence of “false positive” findings decreased its positive predictive value.

Mirroring the results of Figures 7, 8 depicts the results of ADCS of CRC using ^125^I-labeled CC49, the principal primary 2nd-generation mMoAb to TAG-72, for intraoperative detection of DIT in CRC patients [121]. The figure demonstrates that the extent of CRC involves extra-regional tissue in primary and recurrent cases exceeds textbook examples. The surgeon’s exploration using traditional techniques (i.e., visual inspection and palpation) and the HGDP detected the liver’s metastases equally. “The issue is extra-regional tissue.” In this case, the disease involved extra-regional or “non-anatomic” draining lymph nodes that do not follow the usual arterial and venous vessels and, therefore, are unexplored during traditional exploration, including clusters of perirenal, retroperitoneal, and small bowel mesenteric nodes. Occult metastases occurred in the abdominal wall, omentum, pelvic, sacral, and iliac nodes. Many of these lymph nodes are too deep for detection by fluorescence-guided surgery.

A preop roadmap map is needed to ensure that many nodal locations go unexamined today during routine open or MIS procedures. In the case of primary and recurrent CRC, the EODT goes well beyond what traditional surgical approaches explore. We and others have previously reported that assessing EODT using our approach resulted in a change in the extent of surgery in up to a third of primary and recurrent CRC cases using the first and second generation mMoAbs to TAG-72 [115, 122–125] and up to half of primary and recurrent CRC cases.

#### Colorectal Carcinoma as an Exemplar of DIT

What is disease-involved tissue (DIT)? To answer this question, one needs to understand that there is an ongoing debate about what an actual false positive lymph node is, which is HGDP (+) but lacks tumor cells on a single H&E-stained section for routine microscopic examination. The first proof that these lymph nodes are indeed DIT comes from IHC staining demonstrating TAG-72 expression in the germinal centers of these nodes, along with autoradiography demonstrating ^125^I-CC49 similar localization [40]. Some authors say yes, it is a false positive [126–128], while others say not so fast [117, 119, 129–134]. The evidence is conflicting. In a previously reported study of 599 tissue specimens from 92 patients with primary or recurrent CRC [14], we found tumor cells in 134 of 145 (92.5%) non-lymphoid, HGDP (+), and DIT submitted for routine pathology. However, only 71 of the 452 (15.7%) HGDP (+) lymph nodes contained tumor cells on routine H&E-stained slides. These results led us to explore the inherent variables associated with routine pathologic examination of lymph nodes for metastatic tumor cells. Sampling error and detection sensitivity are the two most significant variables. Both are associated with the unequal distribution of tumor cell clusters within a lymph node. Identifying these tumor clusters is helped by using additional slides for H&E and IHC staining; however, they do not eliminate the impact of these variables. Additional sectioning into the lymph node-containing paraffin block and cytokeratin IHC staining identified tumor cells in 102 of 172 (59%) pStage I/II CRC cases. Using a pseudotumor model, we examined the ability of the light microscope to detect tumor cells. We found that cytokeratin IHC staining increased the detection level compared to H&E-stained sections evaluated by three pathologists. It also demonstrated that cytokeratin IHC staining increased microscopic detection sensitivity from <1 tumor cell in 2000 mononuclear cells to <1 in 20,000. Additional detection sensitivity is available using molecular techniques [130–132] and tissue culture [133]. However, the reality is that additional sectioning, cytokeratin IHC staining, molecular genetics, and tissue culture are too expensive and time-consuming for routine use. The actual answer to the clinical importance of pathology-negative HGDP (+) DIT comes from our patient outcome data [117].

#### Clinical Relationship between pStage RO and DIT in ADCS of Colorectal Carcinoma

The literature contains numerous articles that describe various surrogate markers of tumor progression and patient survival. Among these are studies that report that the presence of otherwise undetected occult tumor cells in regional and extra-regional lymph nodes is associated with a worse prognosis for CRC patients [119, 130, 134]. Our ADCS prospective data demonstrates the significant impact of assessing the EODT in patients with either primary or recurrent colorectal carcinoma on their overall survival (Figures 9, 10). The Kaplan-Meier plots demonstrate several significant issues. First, there are two distinct groups of patients with primary or recurrent CRC to which TAG-72 ADCS indicates significant survival variability. Figure 9 demonstrates that in the short term (4-5-year), removing all DIT [i.e., TAG-72 HGDP (+)], regardless of the preferential locator (B72.3 or CC49 mMoAb), provides a significant survival advantage. It also identifies patients who will require adjuvant therapy after surgery Figure 10C depicts the long-term survival of 92 patients with primary CRC injected with I-124 CC49 and CC83 and followed for 125 or more years. Secondly, based on pathologic features, current staging guidelines must include molecular variables. The data in Figure 9 suggest that patient survival can significantly vary from the parochial strict anatomic approaches taken by surgery and pathology alone. Understanding the role of the immune response to TAG-72 and other tumor-associated factors will be critical, and the ability to sample the specific tissues of interest using ADCS will help to facilitate this [14].

### Conclusion

A “systems engineering” concept for assessing the EODT in solid tumors coupled with a multidisciplinary team is essential in today’s management of patients with primary or recurrent disease. Although enrollment of our CRC patients occurred in the late 1980s through the 1990s, the data presented here are one-of-a-kind. It is prospective and includes long-term patient follow-up. Very few studies of TAG-72 expression and ADCS were performed, with few exceptions since we stopped accruing patients. Our studies depended on the use of a family of newly designed handheld gamma probes (Neoprobe, Corp—now Devicor^®^ Medical Products, Cincinnati, OH), a family of newly developed mMoAbs to TAG-72 (provided by Jeffery Schlom, Ph.D., CCR, NCI) and the faculty and staff of The Ohio State University, College of Medicine. Although the data presented here comes from our work, similar significant survival results occurred in a multicenter trial [102]. What is evident from the data in Figures 9, 10 is that there is a critical need for preoperative EODT assessment that provides surgeons and the multidisciplinary team (surgeon, nuclear medicine, and medical oncology) with accurate information upon which to base their clinical decisions. SPECT/CT and PET/CT were unavailable when we conducted our TAG-72 ADCS studies; however, we were frustrated with the preoperative CT imaging that gave us an inaccurate impression of the patient’s EODT.

**FIGURE 11:**
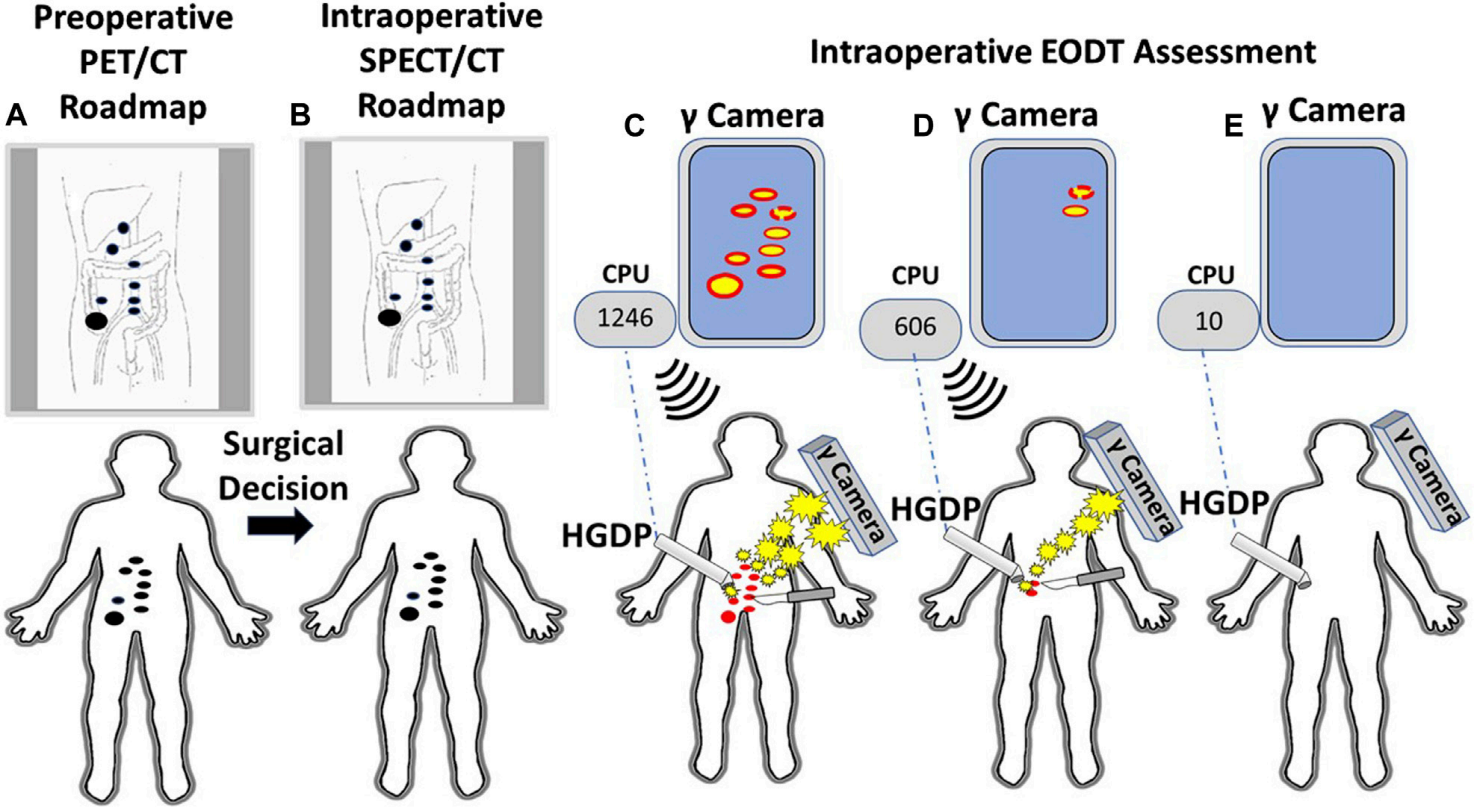
A Systems Engineering Approach to Assessing the Extent of Disease in a Patient with Primary Colorectal Carcinoma (CRC). **(A)** Patient with biopsy-proven CRC receives an intravenous injection of ^124^I-anti-TAG-72 MoAb. A subsequent PET/CT scan demonstrates a right-sided lesion suspicious for CRC (large black dot) with regional and extra-regional disease involved tissue (DIT) (small black dots). A review of the “roadmap” by the clinical team yields a decision for an open approach for surgery with curative intent. **(B)** An intraoperative “roadmap is generated by intravenous injection of ^123^I-anti-TAG-72 MoAb, and SPECT/CT imaging provides an intraoperative EODT “roadmap” (black dots). **(C)** An intraoperative, portable gamma (γ) camera provides the surgeon with real-time images of the DIT (red dots). The surgeon uses the handheld gamma-detecting probe (HGDP) to locate the DIT. The HGDP is connected via Bluetooth (dashed blue line) to a central processing unit (CPU) whose monitor shows the counts per second (cps) of the primary lesion [e.g., 1,246 cps—**(C)**] and DIT 3-sigma above background and an audible signal announces the presence of the DIT that is excised (scapple). **(D)** Real-time intraoperative reimaging demonstrates additional DIT that is then located using the HGDP indicating increased 3-sigma above background signal level (606 cps). The occult DIT is excised (scalpel). **(E)** Before closing of the abdomen, probing of the surgical field shows low background counts (30 cps) on the CPU, and reimaging shows no residual DIT.

The proof-of-concept study involving six cases of gastrinoma used ^111^In-pentetreotide for preoperative and intraoperative imaging using a portable large field-of-view gamma camera and a commercial HGDP [10]. As this study demonstrated, intraoperative EODT assessment requires the simultaneous use of a portable gamma camera and an HGDP. Figure 11 envisions that portion of Figure 1 following the decision for surgery in a case of CRC. This thought experiment uses a currently unavailable PL but current state-of-the-art preoperative and intraoperative imaging technologies and gamma detection probes (e.g., HGDP, laparoscopic, and robotic).

Our proposed concept calls for a portable gamma camera and a gamma detection probe to be used intraoperatively and together rather than in isolation. Bringing instrumentation for intraoperative EODT assessment into the 21st century requires the development of new gamma cameras and probes for open and MIS surgery capable of imaging and detecting “pan-energy” radionuclide tracers. This development is needed because PLs labeled with high energy, short half-life radionuclides allow PET/CT roadmaps to provide preoperative and intraoperative imaging. A single PET/CT generated pre- and intraoperative EODT “roadmap” saves time and money while providing the surgeon and the patient with added value results. However, for EODT to become clinically successful requires continued development of both old and new PLs. PSMA-11 and DOTATATE are our role models for a successful PL for EDOT and would be a great starting place for the clinical approach demonstrated in Figure 11. Clinical grade chimeric and humanized MoAbs, including humanized domain deleted CC49 to TAG-72, are available, and there is ongoing research and development of small fragments for possible EODT assessment. In addition to CRC, MoAbs to TAG-72 are applicable for EODT assessment in most cases of adenocarcinomas of the ovaries, endometrium, breast, prostate, pancreas, stomach, and lungs, opening a new era of multidisciplinary oncology care-based on accurate preoperative and intraoperative EODT imaging and detection [34].

Thirdly, in the case of recurrent CRC (Figures 10A, B), as with primary CRC, TAG-72 ADCS can provide a significant survival advantage to a distinct group of patients. Panel A indicates that we took all comers for our clinical studies [73]. At least 40% (53/131 patients) enrolled were unresectable by ADCS but were addressed by traditional surgery. Another 29 (22%) of patients were found to be unresectable and had HGDP (+) tissue remaining at closing. For the group of 49 patients (37%), there was a significant survival advantage (*p* < 0.0001) as compared to the group of unresectable cases. Panel B depicts the 5-year survival of the 212 primary and recurrent CRC patients who underwent ADCS with either B72.3, CC49, or CC83 mMoAbs over 14 years [38]. Again, we see three distinct populations. The poorest survival is associated with 95 of 212 (45%) cases with unresectable, grossly evident HGDP (+) disease involved tissue remaining at closing, followed by occult HGDP (+) DIT in another 43/212 (20%) of the cases. We see again a subgroup where there is a significant (*p* = <0.0001) survival advantage following the removal of all HGDP (+) tissue. Whereas most studies focus on short-term 5-year survival, Panel C represents a minimum of 15-year survival data from 92 primary CRC patients who underwent ADCS following 125I-CC49 injection [42]. It is important to note that pStage was a significant prognostic variable at each 5-year interval. Although routine pathology is not the best gold standard to judge the impact of EODT assessment, its clinical importance is critical.

Thirdly, in the case of recurrent CRC (Figures 10A, B), as with primary CRC, TAG-72ADCS can provide a significant survival advantage to a distinct group of patients. Panel A indicates that we took all comers for our clinical studies [119]. At least 40% (53/131 patients) enrolled were unresectable by ADCS but were addressed by traditional surgery. Another 29 (22%) of patients were found to be unresectable and had HGDP (+) tissue remaining at closing. For the group of 49 patients (37%), there was a significant survival advantage (*p* < 0.0001) as compared to the group of unresectable cases. Panel B depicts the 5-year survival of the 212 primary and recurrent CRC patients who underwent ADCS with either B72.3, CC49, or CC83 mMoAbs over 14 years [38]. Again, we see three distinct populations. The poorest survival is associated with 95 of 212 (45%) cases with unresectable, grossly evident HGDP (+) disease involved tissue remaining at closing, followed by occult HGDP (+) DIT in another 43/212 (20%) of the cases. We see again a subgroup where there is a significant (*p* = <0.0001) survival advantage following the removal of all HGDP (+) tissue. Whereas most studies focus on short-term 5-year survival, Panel C represents a minimum of 15-year survival data from 92 primary CRC patients who underwent ADCS following ^125^I-CC49 injection [40]. It is important to note that pStage was a significant prognostic variable at each 5-year interval. Although routine pathology is not the best gold standard to judge the impact of EODT assessment, its clinical importance is critical.

Is there a future for our technology? The answer is yes! The future operating room (OR) goals are to provide the surgeon with real-time information designed to enhance the surgical team’s precision, improving patient safety and survival. These changes will improve OR efficiency, leading to more cost-effective solid tumor surgery. A literature search of PubMed for articles written in English from 1984, the year of our first clinical case report results [135] to 2024, —using Medical Subject Heading: image-guided paired with secondary terms including surgery, imaging, fluorescence, and molecular, as well as cancer biomarkers, and gamma camera and probe—showed a plethora of publications, most of which written in the last 10 years, demonstrating continued progress in the development of the technologies critical to our concept.

Our concept of molecular imaging integrates with the technologies associated with computer-assisted minimally invasive surgery (aka, robotic surgery) and image-guided surgery. As such, intraoperative CT and MRI images only provide real-time anatomic data. However, adding intraoperative nuclear molecular imaging using a portable gamma camera will provide location information based on tumor biology independent of tissue location. Fluorescence-guided surgery can be critical for real-time intraoperative assessment of resection margins and superficial occult tumors. Studies are ongoing to look at the ability of dual-labeled preferential locators to assess EODT at the margins and metastatic sites. The next-generation of Bluetooth-enabled gamma probes and fluorescence probes/gamma cameras for intraoperative detection of DIT are under development for both invasive and minimally invasive cancer surgery. Integration of the resulting intraoperative data and its real-time display to the surgeon will require information-bearing monitors throughout the OR. In addition, the surgeon will have a heads-up display that permits viewing of the images and other data without looking up at one of the monitors [136, 137]. Ultimately, the surgeon’s capability to make real-time clinical decisions leads to complete resection of all DIT while reducing the cost of care and improving patient outcomes.

## Figures and Tables

**Table:** 

**ACR**	American College of Radiology
**ADCS**	Antigen-directed cancer surgery
**CH**	Constant heavy chain region of an immunoglobulin molecule
**CEA**	Carcinoembryonic antigen
**CPS**	Counts per second
**CRC**	Colorectal carcinoma
**CT**	Computerized tomography
**EODT**	Extent of diseased tissue
**DIT**	Disease involved tissue
**FAPI**	Fibroblast-activating protein inhibitor
**FDA**	Food and Drug Administration
**FDG**	Fluorodeoxyglucose
**HAMA**	Human anti-mouse antibodies
**H&E**	Hematoxylin and eosin
**HGDP**	Handheld gamma-detecting probe
**IHC**	Immunohistochemical
**kDa**	kilodaltons
**MoAb**	Monoclonal antibody
**mMoAb**	Murine monoclonal antibody
**MRI**	Magnetic resonance imaging
**NCCN**	National Comprehensive Cancer Network
**PET**	Positron emission tomography
**PL**	Preferential locator
**PSMA**	Prostate-specific membrane antigen
**pStage**	Pathologic stage
**RIGS**	Radioimmunoguided surgery
**scFv**	Single-chain fragment variable
**SPECT**	Single-photon emission computed tomography
**TAG-72**	Tumor-associated glycoprotein-72
**TBR**	Tumor-to-Background Ratio (Signal to Noise Ratio)
**VH**	Variable heavy chain of an immunoglobulin molecule
**VL**	Variable light chain of an immunoglobulin molecule

## Data Availability

The datasets presented in this article are not readily available because the data sets dealing with RIGS research are up to 30 years old and no longer are available in any form. The data from the gastrinoma patients is available upon request. Requests to access the datasets should be directed to emerituschuck@gmail.com.
